# A typical carcinoid of the lung – a case report with pathological correlation and propagation of the cancer stem cell line BKZ1 with synaptophysin expression

**DOI:** 10.1097/MD.0000000000018174

**Published:** 2019-12-10

**Authors:** Beatrice Ariane Windmöller, Johannes F.W. Greiner, Christine Förster, Ludwig Wilkens, Fritz Mertzlufft, Jan Schulte am Esch, Barbara Kaltschmidt, Christian Kaltschmidt, Morris Beshay

**Affiliations:** a Department of Cell Biology, University of Bielefeld, Universitätsstrasse 25, Bielefeld; b Institute of Pathology, KRH Hospital Nordstadt, Haltenhoffstrasse 41, Hannover; c Protestant Hospital of Bethel Foundation, Maraweg 21; d Department of General and Visceral Surgery, Protestant Hospital of Bethel Foundation, Schildescher Strasse 99; e Molecular Neurobiology, University of Bielefeld, Universitätsstrasse 25; f Department of General Thoracic Surgery, Protestant Hospital of Bethel Foundation, Burgsteig 13; g Forschungsverbund BioMedizin Bielefeld, FBMB, Maraweg 21, Bielefeld, Germany.

**Keywords:** cancer stem cell, nestin, neuroendocrine tumors, synaptophysin, typical carcinoids

## Abstract

**Rationale:**

Neuroendocrine tumors (NETs) of the lung account for 5% of all cases of lung cancer, which itself is the leading cause of cancer-related death worldwide. In accordance to its rarity, only few cell lines of NETs exist, which even often lack key characteristics of the primary tumor, making it difficult to study underlying molecular mechanisms.

**Patient concerns:**

The patient reported in this case is a 71-year old woman, which never smoked but suffered under dry cough.

**diagnoses:**

Chest CT-scan showed a paracardiac nodule of the lingula with 2 × 1.8 cm in diameter.

**Interventions:**

The detected paracardiac nodule of the lingula was anatomically resected using video assisted thoracic surgery.

**Outcomes:**

Histopathological diagnostic of the removed tissue identified the tumor as a well-differentiated typical carcinoid (TC), which represents one of the four subgroups of pulmonary NETs. Next to the successful treatment of the patient, we were able to propagate cancer stem cells (CSCs) out of the resected tumor tissue. To the best of our knowledge, we firstly isolated CSCs of a typical carcinoid, which were positive for the prominent CSC markers CD44, CD133 and nestin, confirming their stem cell properties. Additionally, CSCs, further referred as BKZ1, expressed the neuroendocrine marker synaptophysin, verifying their neuroendocrine origin. However, nuclear synaptophysin protein was also present in other stem cell populations, suggesting a role as general stem cell marker.

**Lesson:**

In line with the importance of CSCs in cancer treatment and the lack of CSC-models for neuroendocrine neoplasms, the here described BKZ1 cancer stem cell line of a typical carcinoid represents a promising new model to study pulmonary carcinoids and particular NETs.

## 1 Introduction

Lung cancer is the leading cause of cancer-related death worldwide, with about 34,500 new cases of male and 19,300 new cases of female annually in Germany. The relative overall 5-year survival rate, is 15% in males and 20% in females.^[1]^ Neuroendocrine tumors (NETs) of the lung are rare with only 5% of all newly diagnosed malignancies.^[2,3]^ Although the lungs are the second most common site of origin for neuroendocrine tumors especially for typical carcinoid after the gastrointestinal tract,^[4]^ it has been reported to develop even synchronously in both lungs.^[5]^ In the lung, NETs derive from solitary pulmonary neuroendocrine cells (PNECs) or from aggregated PNEC clusters (neuroepithelial bodies),^[6–8]^ which initially act as the stem cell niche.^[9,10]^ PNECs gain various mutations during carcinogenesis, which are responsible for the dedifferentiation into high tumorigenic cancer stem cells (CSCs).^[11]^ Based on the capacity for self-renewal and differentiation as well as their invasiveness and resistance to chemotherapy, CSCs are crucial mediators of metastasis, cancer relapse or immune system escape and are thus of enormous clinical interest.^[12–14]^

In the current World Health Organization (WHO) classification 2015 lung NETs are categorized into four histologic variants defined as well differentiated, low-grade typical carcinoid (TC), well-differentiated, intermediate-grade atypical carcinoid (AC), slightly differentiated, high-grade large cell neuroendocrine carcinoma (LCNEC) and slightly differentiated, high-grade small cell lung carcinoma (SCLC).^[15,3]^

The group of well-differentiated lung NETs comprise approximately 27% of all NETs^[16]^ and develop in non or current light smokers.^[17]^ Moreover, TCs and ACs are capable of lower mitotic rates, necrosis and genetic abnormalities in comparison to high-grade NETs.^[18]^ Although well-differentiated NETs are not that aggressive, the incidence increased over the last 30 years about 6% annually,^[19]^ with TCs being the more frequent form of well-differentiated NETs.^[20]^ Here, we report a case of a typical carcinoid (TC) of the upper lobe of the left lung as well as the successful *in vitro* propagation and characterization of cancer stem cells out of the resected tumor tissue.

## 2 Case report

**Figure 1:**
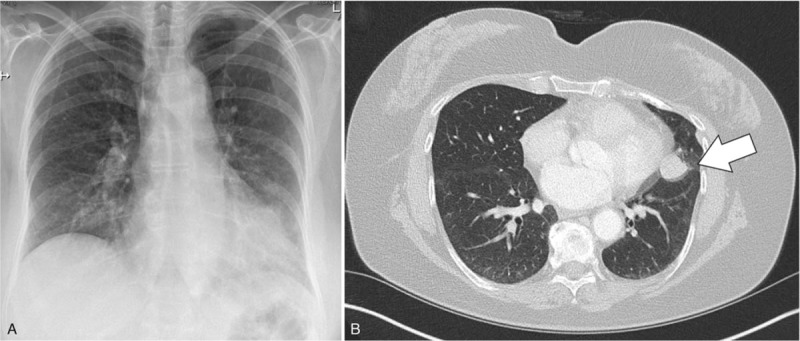
Radiological examination of the chest of the patient. (A) Radiograph of the chest revealed an uneven mass in the left lung. (B) Chest CT scan displayed a paracardiac tumor (arrow).

A 71-year old woman was admitted to the hospital in November 2018. The clinical examination showed no abnormalities. Biochemical parameters in blood showed normal values apart from slightly elevated gamma-glutamyl-transferase (GGT) (160 U/l, normal up to 40U/l). She never smoked and had no family history of lung or gastrointestinal cancers. She developed dry cough over the last 6 weeks, which was resistant to treat. Therefore a chest X-ray was done, which showed an irregular left border of the heart. A subsequent chest CT-scan showed a paracardiac nodule with 2 × 1.8 cm in diameter (Fig. 1), no mediastinal lymph nodes enlargement and no pleural effusions were detected.

A bronchoscopic examination with bronchial lavage was done. The lavage revealed acid proof rods, which were immediately tested for *M. tuberculosis* by quantiferon screening. Since the medical report was negative for tuberculosis, surgery was performed for histological diagnosis.

**Figure 2:**
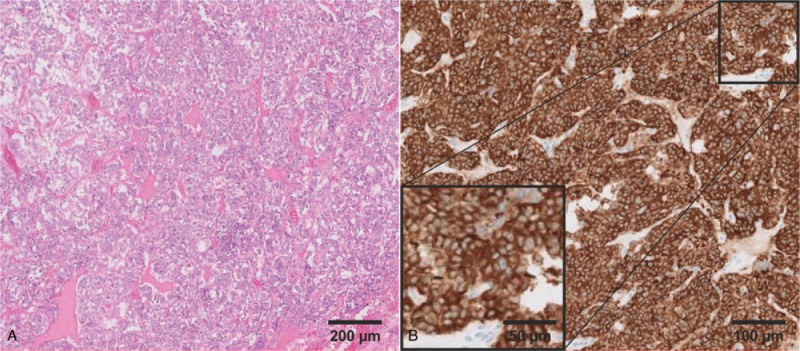
Histopathological analysis of the neuroendocrine tumor tissue. (A) Hematoxylin and eosin staining revealed a well differentiated neuroendocrine morphology, characteristic for typical carcinoids. (B) Moreover, tissue was positive for the neuroendocrine marker synaptophysin.

The exploration of the entire hemithorax left showed massive dorso-basal adhesions between the lower lobe and the thoracic wall, as well as the diaphragm. After adhesiolysis, the tumor within the lingual segment was exposed, biopsied and a histopathological frozen section examination was performed, which showed malignancy. The tumor was then anatomically resected using video assisted thoracic surgery (VATS) to remove both segments of the lingual. Complete mediastinal lymph node dissection was done. Histopathological analysis of the removed tissue indicated a neuroendocrine neoplasm, which was confirmed by immunohistochemistry. In particular, cancerous tissue was positive for synaptophysin (Fig. 2), chromogranin A as well as high and low molecular weight cytokeratins detected by the antibody combination of AE1/AE3. Based on the absence of the epithelial marker TTF1 as well as the neuroendocrine markers CDX2 and cytokeratin 20 of the gastrointestinal tract, LCNEC, SCLC or a metastasis of the gastrointestinal tract could be excluded. Neither an apparent necrosis within the tissue, nor pathologic lymph node structures were observed. Further analysis revealed only 1% to 2% Ki67-positive mitotic cells within the tumor, resulting in the final classification of a well-differentiated, low-grade typical carcinoid (TC) in stage IA. Therefore, no adjuvant therapy was suggested. The patient was discharged on the fifth day after surgery in a good general condition. 6 months follow up showed no abnormalities. The somatostatin receptor imaging with ^68^G DOTATATE PET/CT- showed no abnormal findings.

**Figure 3:**
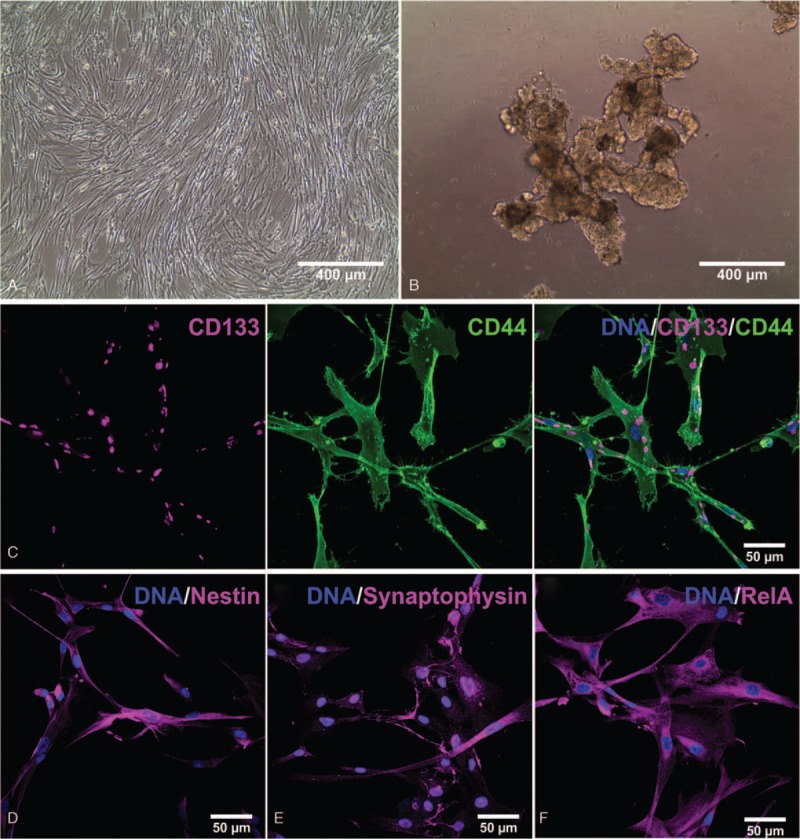
Successful isolation and characterization of cancer stem cells out of the tumor tissue of a typical carcinoid. (A) Cancer stem cells were grown as adherent culture within stem cell media supplemented with 10% fetal calve serum (FCS), (B) as well as sphere culture in a low attachment flask without FCS as supplement. (C) Immunocytochemical staining for the stem cell markers CD133 and CD44, revealed double positive cells, confirming the isolation of cancer stem cells. Additionally, cells were positive for the stem cell and primitive neuroectoderm marker (D) nestin, the neuroendocrine marker (E) synaptophysin, and the (F) NF-κB subunit p65.

Next to a histopathological analysis, a part of the resected typical carcinoid was used for the attempt to cultivate and characterize cancer stem cells. Informed consent according to local and international guidelines was signed and all further experimental procedures were ethically approved (Ethics committee Münster, Germany, 2017–522-f-S). For the isolation of the CSCs the specimen was washed twice with ice-cold phosphate buffered saline (PBS), mechanically disintegrated in 2 to 5 mm pieces followed by an enzymatically digestion with collagenase for 2 hours at 37°C. One half of the minced tissue was used to cultivate spheres in Dulbecco modified Eagle's medium/Ham's F-12 with addition of 200 mM L-Glutamin, epidermal growth factor (EGF; 20 ng/mL), basic fibroblast growth factor (bFGF/FGF-2; 40 ng/mL) and B27 supplement in low adhesion T25 tissue culture flasks (Fig. 3B). The other half of the tissue was used to grow adherent CSCs, where the cells were cultivated on gelatin coated culture dishes in the medium described above supplemented with 10% fetal calf serum (Fig. 3A).

**Figure 4:**
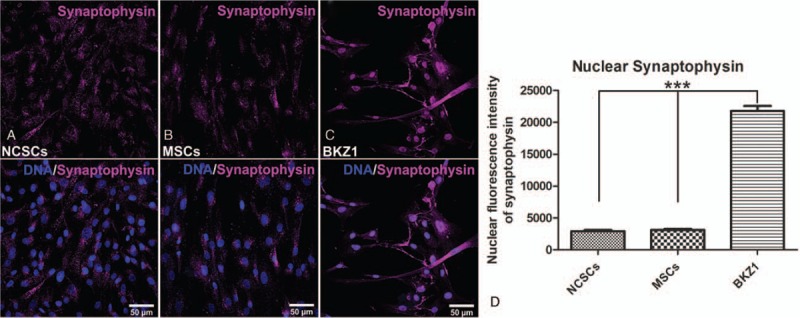
Nuclear synaptophysin expression of BKZ1 and different non-pathogenic stem cells. Immunocytochemical staining of synaptophysin in female donor-derived (A) human neural crest-derived stem cells (NCSCs),^[21,22]^ (B) adipose tissue-derived mesenchymal stem cells (MSCs) isolated according to Solemani and Nadri^[23]^ and (C) the here described neuroendocrine lung cancer stem cells (CSCs). (D) Quantification of the nuclear fluorescence intensity of synaptophysin within the different stem cell populations. Means ± SD was analyzed by one way ANOVA test (Kruskal-Wallis statistic). *P* < .05 was considered as statistically significant. Analysis of data was done by using GraphPad Prism 5.00 software (San Diego, CA).

After successful cultivation, cells were analyzed according to their expression profile of cancer stem cell and neuroendocrine specific markers, as well as their morphology. Immunocytochemical double staining of the lung cancer stem cell markers CD133 and CD44 confirmed the isolation of cancer stem cells (Fig. 3C). Additionally, cultivated cells were positive for the neuroendocrine marker synaptophysin underscoring the establishment of the relevant cancer stem cells. Synaptophysin was especially localized within the nucleus of the cells, although some cells also revealed synaptophysin within their cytoplasm (Fig. 3E). Next to the expression of synaptophysin in the isolated neuroendocrine cancer stem cells (Fig. 3E, 4C), we detected synaptophysin in neural crest-derived stem cells from the nasal cavity of a female donor^[23,22]^ (Fig. 4A) and female adipose tissue-derived mesenchymal stem cells (Fig. 4B), suggesting a new role of synaptophysin as a stem cell marker. Quantification of the nuclear fluorescence intensity of synaptophysin within the different stem cells revealed a significant higher expression within the isolated BKZ1 cell line in comparison to non-pathogenic stem cells (Fig. 4D). Furthermore, cells expressed the primitive neuroectoderm and stem cell marker nestin, underlining the stem cell characteristics and suggesting a neural crest origin of the cultivated cells (Fig. 3D). Due to the strong association of NF-κB with chronic inflammation and different cancer types, TC-derived BKZ1 cells were analyzed according to their NF-κB expression. Immunocytochemical staining of the subunit RELA (p65) displayed a high perinuclear expression of the cultured cells (Fig. 3F).

## 3 Discussion

Neuroendocrine lung tumors can be divided into four histological groups, comprising typical carcinoid (TC), atypical carcinoid (AC), large cell neuroendocrine carcinoma (LCNEC) and small cell carcinoma (SCC).^[15]^ Although the distinction between the different neuroendocrine lung tumor types is possible by a good histopathological analysis, the diagnosis of a neuroendocrine lung tumor at all is difficult.^[18]^ Symptoms of lung NETs are often nonspecific or absent, leading to delays in diagnosis. Moreover, clinical data regarding lung NETs are rare, especially for carcinoids, making diagnosis and treatment difficult.^[24]^ This problem is also described by a recently published global survey, which collected data on NETs from a patient's perspective, revealing that the diagnosis of lung NETs of 50% of the 222 patients lasts up to 2 years.^[25]^ Moreover, the incidence of the carcinoids, especially TCs, increased over the last 30 years about 6% annually in contrast to the incidence of SCC.^[19]^ Furthermore, they possess the earliest occurrence age on average within the group of NETs with 45 years.^[18,26,27]^ Treatment of choice for localized TCs is the anatomical resection of the tumor,^[18]^ with 5 to 10-year survival rates higher than 90%,^[28]^ whereas the use of adjuvant therapy is usually not recommended for TCs due to rare involvement of the lymph nodes.^[18]^ Even if TCs are known as low-grade NET, 5% to 20% of TCs metastasize, preferentially to the liver or bone.^[29]^

Based on the increased incidence, the difficult diagnosis, and the limited knowledge about the underlying molecular mechanisms of TCs, it is important to establish a good model for studying the biology of this tumor type. Currently there are only few TC cell lines available. Moreover, many of those cell lines failed to generate xenograft tumors, displaying the absence of cancer stem cells within the cell population.^[30]^ We now present a successful isolation of cancer stem cells out of the tissue of this rare tumor type, which enables new opportunities to investigate the molecular mechanisms of resistance to conventional chemotherapeutics, biological molecules, targeted therapies and radiotherapy, which are known to be caused by cancer stem cells. Initial characterization of the isolated CSCs, revealed a high expression of the cancer stem cell marker CD44, which is known to mediate cancer cell survival, proliferation and motility, as well as the modulation of tumor microenvironment.^[31,32]^ Moreover, it is known that CD44 expression is dominant within pulmonary carcinoids, decreasing from TC to AC to very low levels in LCNEC and SCLC,^[33,34]^ which stands in line with the presented data. Moreover, isolated CSCs expressed the cell surface glycoprotein and CSC marker CD133, which is linked to poor prognosis in NSCLC.^[35]^ Additionally, CD133 positive cells are known to have significantly higher abilities of self-renew, drug resistance and tumor initiation.^[36]^ Although, concerning the expression of CD133 within typical carcinoid cell lines only less is known, which can be explained by the failure to isolate CSCs by other groups, Sakai et al. showed that 18% of well-differentiated pancreatic neuroendocrine tumors are CD133 positive.^[37]^ Due to the identification of nestin as CSC marker in NSCLC,^[38]^ its protein expression was investigated and highly detected within the here presented isolated CSCs. Furthermore, nestin is known as primitive neuroectoderm^[39]^ and neural stem cell^[40]^ marker, which suggests a possible correlation between pulmonary typical carcinoids and the neural fate. Within a retrospective evaluation of 88 patients with neuroendocrine lung tumors using immunhistochemistry, nestin was detected in 17% of specimens, being a negative prognostic factor and significantly higher expressed in LCNEC in comparison to carcinoids.^[41]^ This suggests that the CSC amount increases from TC to AC and LCNEC to SCC, indicated by a higher expression of nestin, leading to a decrease in the survival rate of the patient. Moreover, isolated CSCs showed a high amount of synaptophysin protein, underlying their neuroendocrine origin.^[42]^ Assuming nuclear localization of synaptophysin as a general stem cell characteristic, we demonstrated the appearance of nuclear synaptophysin protein also in non-pathogenic human stem cell populations like NCSCs and MSCs. However, the nuclear expression of synaptophysin of the CSCs was significantly higher in comparison to the other stem cells, which may be due to their cancerous origin.^[43]^ Next to stemness related proteins, the isolated CSCs expressed the NF-κB subunit RelA. NF-κB is involved in multiple steps in carcinogenesis and in cancer cell's resistance to chemo- and radio-therapy. Moreover, tumor samples obtain from lung cancer patients revealed high levels of NF-κB activation, which was significantly associated with poor prognosis and tumor stage.^[44,45]^ This is in accordance with a meta-analysis concerning the prognostic significance of NF-κB expression in NSCLC, where Gu et al. showed that high NF-κB expression is positively associated with poor survival outcome of NSCLC patients, suggesting a tumor promotive function of NF-κB. Additionally, they presented a positive correlation of NF-κB with tumor stage and lymph node metastasis.^[46]^ Concerning the particular effect of RelA, Chen et al. could show its influence on the sensitivity of NSCLC to paclitaxel, which was increased by the knockout of NF-κB p65.^[47]^ Furthermore, Khan et al. linked the anti-cancer efficacy of curcumin, to its HIF-1α and RelA decreasing activity in lung cancer cells.^[48]^ Regarding neuroendocrine lung tumors NF-κB is known to play a crucial role regulating tumor cell proliferation and resistance to apoptosis.^[49,50]^ Furthermore, Shao et al. displayed that 55.6% of neuroendocrine tumors were positive for the leukemia related protein 16 (LRP16), which is an important estrogen-responsive gene and a crucial regulator for NF-κB activation, suggesting a proliferative effect due to the activation of NF-κB pathway.^[51]^

In conclusion, cancer stem cells were for the first time to our knowledge successfully isolated out of a typical carcinoid of the lung, representing a promising model to study the underlying molecular mechanism and possible treatment strategies for this rare tumor type.

## Acknowledgments

The excellent technical assistance of Angela Kralemann-Köhler, Claudia Rose and Ulrike Hormel is gratefully acknowledged.

## Author contributions

**Conceptualization:** Barbara Kaltschmidt, Christian Kaltschmidt, Morris Beshay.

**Data curation:** Beatrice Ariane Windmöller, Barbara Kaltschmidt, Christian Kaltschmidt, Morris Beshay.

**Formal analysis:** Beatrice Ariane Windmöller, Morris Beshay.

**Investigation:** Beatrice Ariane Windmöller, Johannes F. W. Greiner, Christine Förster, Ludwig Wilkens, Fritz Mertzlufft, Jan Schulte am Esch, Barbara Kaltschmidt, Christian Kaltschmidt, Morris Beshay.

**Writing – original draft:** Beatrice Ariane Windmöller, Johannes F.W. Greiner.

Beatrice Ariane Windmöller orcid: 0000-0002-5918-9384.
